# Influence of Magnesium Ions in the Seawater Environment on the Improvement of the Corrosion Resistance of Low-Chromium-Alloy Steel

**DOI:** 10.3390/ma11010162

**Published:** 2018-01-20

**Authors:** Sol-Ji Song, Jung-Gu Kim

**Affiliations:** School of Advanced Materials Science and Engineering, Sungkyunkwan University, 300 Chunchun-Dong, Jangan-Gu, Suwon 440-746, Korea; mysolgi@gmail.com

**Keywords:** low-alloy steel, EPMA, electrochemistry, corrosion, rust layer

## Abstract

This study examined the synergic effect of alloying the element Cr and the environmental element Mg^2+^ ions on the corrosion property of a low-alloy steel in seawater at 60 °C, by means of electrochemical impedance spectroscopy (EIS), linear polarization resistance (LPR) tests and weight-loss tests. The Mg^2+^ ions in seawater played an important role in lowering the electron transfer of the rust layer in the Cr-containing steel. The corrosion resistance of the Cr-containing steel is superior to that of blank steel in Mg^2+^ ions containing seawater. XPS and XRD results indicated that the formation of MgFe_2_O_4_ and a mixed layer (Cr oxide + FeCr_2_O_4_ + MgCr_2_O_4_) improved the corrosion resistance of the low-alloy steel in the seawater.

## 1. Introduction

The water ballast tank (WBT) is a structure that is used for controlling vessel weight with seawater to provide vessel balance and stability. The repetition of the seawater inflow and outflow induces the exposure of the WBT to various marine environments, and the seawater acts as a corrosion factor, due to the various aggressive ions and organisms [1,2,3,4,5].

In recent years, numerous studies have attempted to improve the service life of the WBT. The primary focus of these studies comprises the following two parts: coating technologies and corrosion-resistant alloy designs. A zinc (Zn) epoxy-primer coating is mainly used in the ballast tank for the prevention of corrosion; however, this coating is not an ideal protector of the ballast tank because of existing defects, and therefore high-corrosion-resistant steel is required [6,7,8,9,10,11]. Many studies on the alloying elements in carbon steels have been undertaken because it is widely used in seawater structures due to its high availability, simple fabrication process, and low cost [12,13,14,15,16,17,18,19,20].

The typical alloying elements that are used to increase the corrosion resistance are not only chromium (Cr), aluminum (Al), and nickel (Ni), but a combination of the three elements is also used. In particular, regarding the effect of Cr in the high-strength low-alloy (HSLA) steels that are reported by Blekkenhorst et al. [21], it was mentioned that, as an alloying element, Cr is beneficial for general corrosion. Furthermore, Zhang et al. [22] and Yamashita et al. [23] reported that Cr promotes the formation of *α*-Cr_x_Fe_1-x_OOH, called Cr-goethite, and this compact rust layer protects the matrix from chloride anions by changing the surface property under the cation-selectivity condition.

The corrosion of carbon steel is influenced by various seawater constituents. The chloride and sulfate ions are representative anions that cause serious corrosive problems. The major cations that contribute to the corrosion property are calcium (Ca) and magnesium (Mg) ions, as they produce local alkaline surface conditions, due to the precipitative effects of calcium carbonate (CaCO_3_) and magnesium hydroxide (Mg(OH)_2_). CaCO_3_ and Mg(OH)_2_ are attributed to the cathodic reduction of oxygen (O). The deposits impede the transport of dissolved O to the metal surface and eventually decrease the corrosion rate. The precipitate formations are derived according to the following reactions [24,25]:

Ca^2+^ + HCO_3_^−^ + OH^−^ → CaCO_3_ + H_2_O (1)

Mg^2+^ + 2OH^−^ → Mg(OH)_2_ (2)

Although the research regarding these deposits has received much attention [25,26], little attention has been paid to the relationship between the alloy elements and the seawater cations, in terms of corrosion resistance. Thus, the purpose of this study is the evaluation of the relationship between the Cr-alloying element and the seawater cation in synthetic seawater, for which electrochemical tests and surface analyses were used.

## 2. Results and Discussion

### 2.1. Interaction between Cr and Seawater Cation

**Figure 1:**
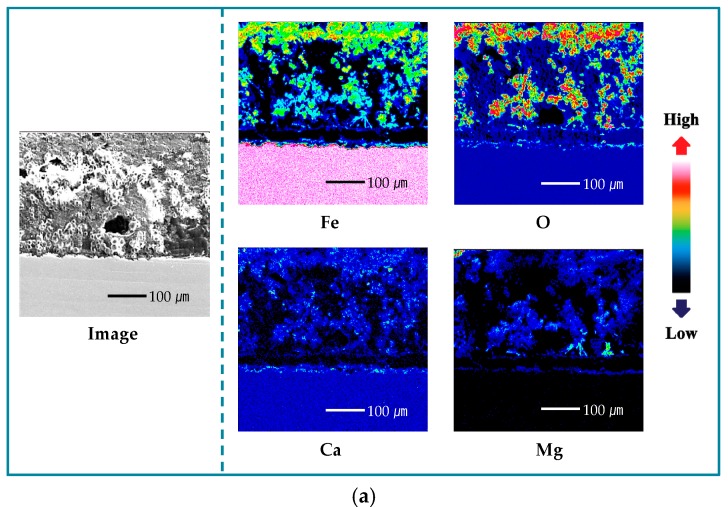

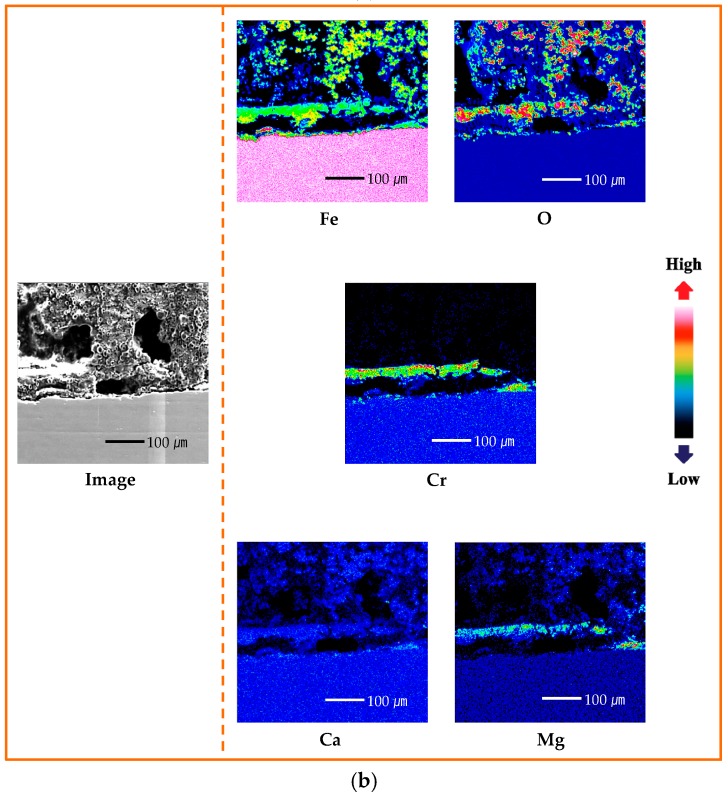
Electron probe microanalyzer (EPMA) cross-sectional mapping results of specimens after 30 days immersion in seawater with Mg^2+^ ions: (**a**) blank steel and (**b**) 0.7-wt.%-Cr steel.

To identify the distribution and the concentration of the alloying elements, the rust layer of the specimens that were immersed in seawater for 30 days were analyzed using an electron probe microanalyzer (EPMA). Figure 1 shows the cross-sectional EPMA-mapping results for blank and Cr-alloying steels. The distribution of iron (Fe) is nonuniform, and the concentration is lower than that of the substrate, indicating that a part of the Fe in the rust layer had dissolved and the Fe-forming rust layer was produced by dissolution of the substrate. Also, the Fe in the Cr-alloying steel is distributed more closely to the substrate, compared with that of the blank steel. Due to the higher distribution and concentration of O compared with the substrate, oxide and hydroxide were formed in the rust layer. The localization of Cr in the rust layer near the substrate suggests that the alloying element, Cr, forms an enrichment layer, which is expected to improve corrosion resistance [21,22,23,27].

In regard to the seawater cation, Ca is distributed in the rust layers on both the blank- and 0.7-wt.%-Cr steel rust layers. Mg, however, showed a different trend in both of the specimens, as follows: It is distributed throughout the rust layer on the blank steel, but it is condensed in the 0.7-wt.%-Cr steel; furthermore, the concentrated-Mg region is included in the Cr-enrichment layer. From this result, it was expected that both the Mg and the Cr would contribute to the corrosion resistance. Thus, to identify the Cr–Mg synergic effect, the experiment was conducted in both the presence and the absence of the Cr-alloying element and the Mg^2+^ ions, in seawater.

### 2.2. Corrosion Rate Calculation

The corrosion rates of the specimens after 30 days of immersion were calculated using electrochemical impedance spectroscopy (EIS) and the linear polarization resistance (LPR) test. By fitting the EIS data and using the slopes of the potential and the current, the polarization resistance (*R_p_*) can be obtained from the EIS and LPR data. Then, the *R_p_* is transferred to the corrosion-current density, using the following equation [28]:

(3) $$R_{p}=\frac{\beta_{a}\beta_{c}}{2.3\;i_{corr}\;(\beta_{a}+\beta_{c})}$$

where $\beta_{a}$ and $\beta_{c}$ are the anodic and cathodic Tafel slopes, respectively, and *i_corr_* is the corrosion current density (μA/cm^2^). The impedance parameters of each specimen, including the *R_p_*, are listed in Table 1. The corrosion rate can be determined from the *i_corr_* through Faraday’s law [28], as follows:

(4) $$Corrosion\;rate(mm/y)=\frac{3.16\times 10^{2}\times i_{corr}\times M}{zF\rho }$$

where *M* is the molar mass of the metal (g/mol), *z* is the number of transferred electrons per metal atom, *F* is Faraday’s constant, and *ρ* is the metal density (g/cm^3^). 

**Figure 2:**
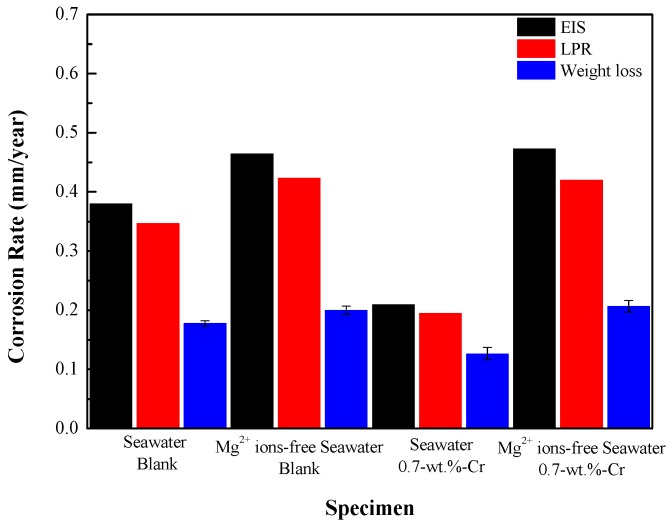
Corrosion rates of specimens, measured by electrochemical impedance spectroscopy (EIS), linear polarization resistance (LPR) and weight-loss measurements for 30 days.

Also, the weight loss-calculated corrosion rate was determined using the following equation [28]:

(5) $$Corrosion\;rate(mm/y)=\frac{87,600\;W}{At\rho }$$

where 87,600 is the metric- and time-conversion factor, *W* is the weight loss (g), *A* is the exposure area (cm^2^), *t* is the immersion time (h), and *ρ* is the density (g/cm^3^). Figure 2 shows the corrosion rates of the blank and 0.7-wt.%-Cr steels after the 30-day immersion in seawater, with and without the Mg^2+^ ions. The corrosion rates calculated by EIS, LPR, and weight loss tests showed the similar trend in all three experiments: The average corrosion rates of the steels decreased in the following order: 0.7-wt.%-Cr steel without the Mg^2+^ ions ≈ blank steel without the Mg^2+^ ions > blank steel with the Mg^2+^ ions > 0.7-wt.%-Cr steel with the Mg^2+^ ions. This result shows that the Cr-alloying element only improved the corrosion resistance in the seawater containing the Mg^2+^ ions. The difference in the corrosion rates of the blank steel, in the presence and absence of the Mg^2+^-containing seawater, is due to the Mg(OH)_2_ that acts as a physical barrier. In the 0.7-wt.%-Cr steel, the difference in corrosion rate between the Mg^2+^-containing and Mg^2+^-free seawater solutions is larger than that of the blank steel, which can be explained by not only the Mg^2+^-containing effect, but also the barrier effect between the Mg^2+^, Fe^2+^, and Cr^3+^ ions. As shown in Figure 1b, it is expected that the Mg^2+^, Fe^2+^, and Cr^3+^ ions that are concentrated near the substrate will form a compound and act as a barrier against corrosion.

### 2.3. Electrochemical Impedance Spectroscopy (EIS)

**Figure 3:**
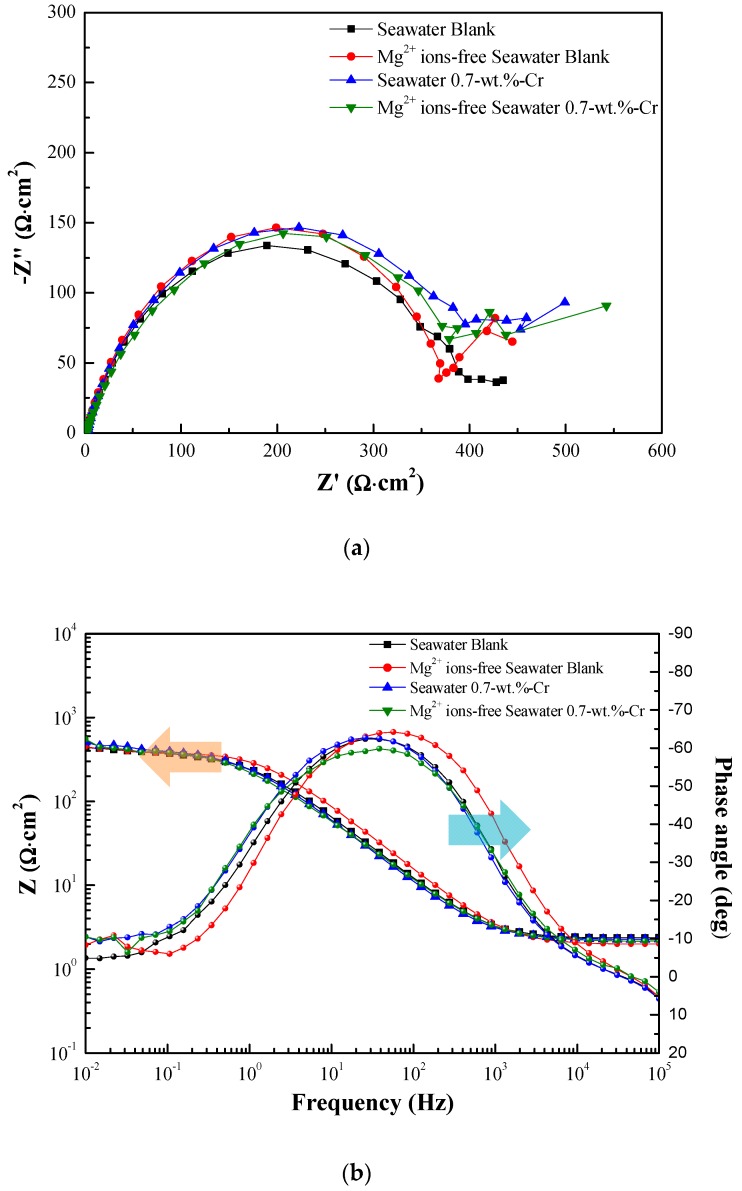

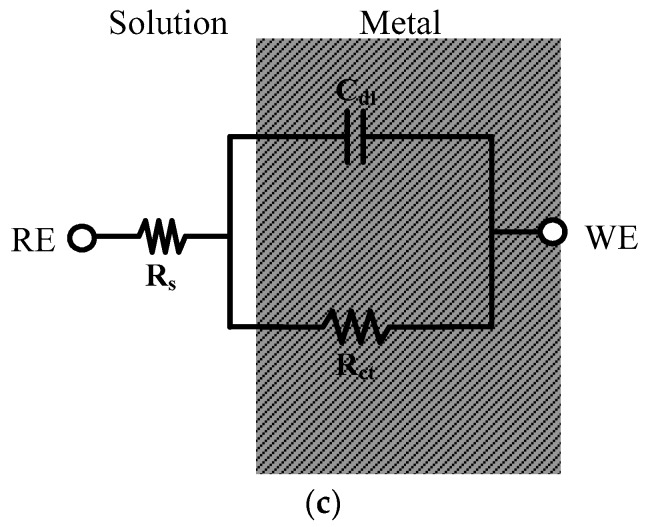
Impedance spectra data of specimens immersed in 60 °C seawater for 1 day: (**a**) Nyquist plots, (**b**) Bode plots and (**c**) equivalent circuit.

The Nyquist and Bode plots of the specimens after 1 day of immersion are shown in Figure 3a,b. Since the formation of the rust layer is insignificant, the impedance results can be fitted to the circuit model that is shown in Figure 3c, as one time constant, and the impedance parameters that are listed in Table 1.

The equivalent circuit consists of the following elements: R_s_ represents the solution resistance, C_dl_ is the capacitance that is generated by the electric double layer, and R_ct_ is the charge-transfer resistance. The RE is the reference electrode (saturated calomel electrode), and WE is the working electrode (specimens). The experiment results show that the *R_p_* values are similar, regardless of the presence of the Cr-alloying element and the Mg^2+^ ions in the seawater.

**Figure 4:**
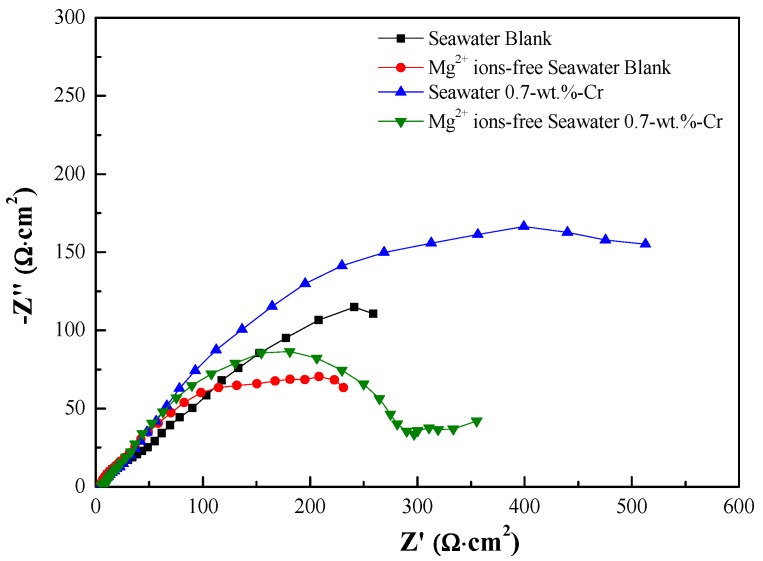
Nyquist plots for specimens immersed in 60 °C seawater for 30 days.

**Figure 5:**
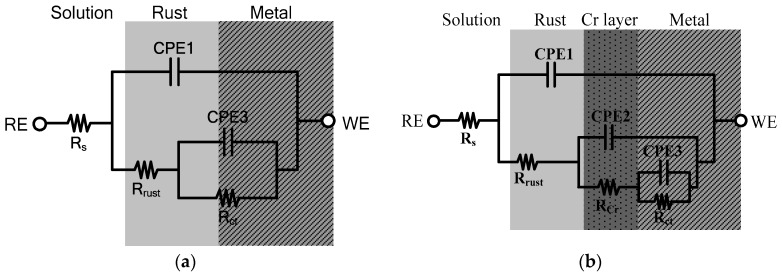
Equivalent circuit models for interpretation of the impedance spectra after 30 days: (**a**) blank steel and (**b**) 0.7-wt.%-Cr steel.

Also, the Nyquist plot impedance spectra after the 30 days of immersion are shown in Figure 4. According to the different rust layers, the equivalent electrical circuits were used to fit the results of the EIS tests, as shown in Figure 1. Figure 5 shows each of the equivalent electrical circuits of the blank and 0.7-wt.%-Cr steels. Due to the Cr-enrichment layer, the spectra of the 0.7-wt.%-Cr steel exhibited a three-time constant, while the blank steel exhibited a two-time constant. The equivalent circuit consists of the following elements: R_s_ represents the solution resistance, C_rust_ is the rust capacitance, R_rust_ is the rust resistance, C_dl_ is the capacitance that is generated by the electric double layer, and R_ct_ is the charge-transfer resistance. The RE is the reference electrode (saturated calomel electrode), and WE is the working electrode (specimens). Also, C_Cr_ is the capacitance that is formed by the Cr-enrichment layer and R_Cr_ is the Cr-enrichment-layer resistance. In this instance, the capacitors were replaced with the constant phase elements (CPEs) for more accurate EIS-data fitting, where not only a double layer capacitance (C) but also a phenomenological coefficient (*n*) have been included. The CPE impedance takes the following form:

(6) $$Z_{\mathrm{CPE}}=\;\frac{1}{Q{\left(j\omega \right)}^{n}}$$

where *Q* is an effective CPE coefficient, *ω* is the sine-wave-modulation angular frequency, *j* is an imaginary number, and *n* is the phenomenological coefficient that characterizes the phase shift [29,30,31,32]. Thus, in this paper, C_rust_, C_Cr_, and C_dl_ were replaced with CPE1, CPE2, and CPE3, respectively.

In the Nyquist plot, the degree of depression of the semicircle, with a center below the real axis, determines the depression angle (*α*). The depression angle is an empirical factor that represents the deviation from the ideal capacity [33,34]. The increase in the depression angle means there is an increase in surface inhomogeneity, due to surface roughness and layer porosity [35,36]. The depression angle that is calculated by the following equation is associated with the phenomenological coefficient (*n*) [29,32,33]:

(7) $$\alpha \;=(1\;-\;n)\;\times 90^{\circ }$$

Table 1 lists the fitted EIS data from the 1-day and 30-day immersions, which determined the optimized values that were obtained using the ZSimpwin program (Princeton Applied Research, USA), according to each of the equivalent electrical circuits in Figure 5 [37,38]. The total polarization resistance (*R_p_*) that is represented in Table 1 is equal to R_rust_ + R_ct_ for the blank steel and R_rust_ + R_Cr_ + R_ct_ for the 0.7-wt.%-Cr steel. In the 0.7-wt.%-Cr steel, the calculated depression-angle value of the Mg^2+^-containing seawater specimen is 17.433° and that of the Mg^2+^-free specimen is 21.186°. This result means that the characteristics of the Cr-enrichment layers that formed on both specimens are different, as follows: The 0.7-wt.%-Cr steel with the Mg^2+^ ions comprises a dense layer compared with that without the Mg^2+^ ions. Also, the depression-angle values of the blank steel are 21.213° and 22.833°, with and without the Mg^2+^ ions, respectively. This porosity difference, regarding the rust layer, is due to the physical barrier that is caused by the Mg^2+^ ions in the seawater, which also causes the corrosion rate difference for the blank steel.

**Figure 6:**
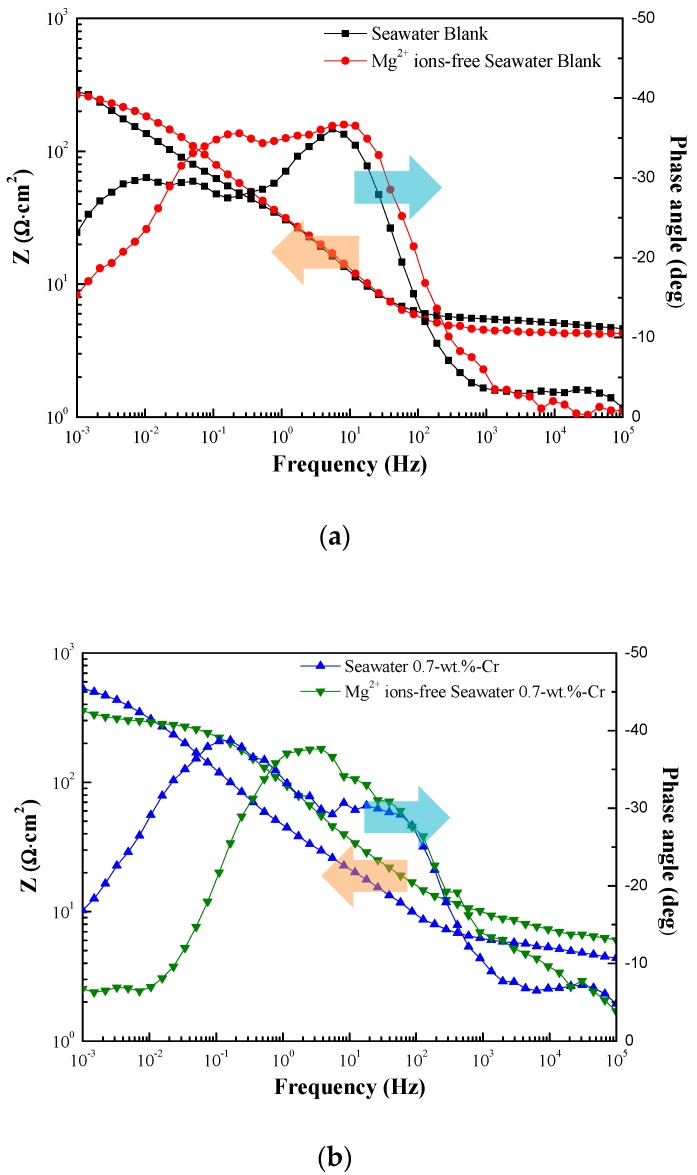
Bode plots for the specimens immersed in 60 °C seawater for 30 days. Specimens are (**a**) blank steels, with and without Mg^2+^ ions and (**b**) 0.7-wt.%-Cr steels with and without Mg^2+^ ions. The splitting of the phase angle to lower frequency means the change of capacitance.

Figure 6 shows the EIS Bode plots of the blank and 0.7-wt.%-Cr steels, with and without the Mg^2+^ ions, for the 30-day immersion in the synthetic seawater at 60 °C. The splitting of the phase angle frequency curve indicates the presence of other phases.

In Figure 6, each of the split shoulders on the phase angle are shifted to the lower frequency, compared with the Mg^2+^-free seawater, meaning that the capacitance values of the Mg^2+^-containing seawater are larger, as identified in Table 1. In the blank steel, the C_rust_ value is larger in the Mg^2+^-containing seawater. Because the capacitance is inversely proportional to the thickness, the rust layer in the Mg^2+^-free seawater is thicker, due to the greater corrosion. Also, the C_dl_ value, which is formed as the ions are absorbed onto the electrode surface, is larger in the Mg^2+^-containing seawater. Thus, the different values are reasonable because the Mg^2+^-containing seawater comprises a higher quantity of adsorbable ions. In the 0.7-wt.%-Cr steel, the capacitance values—C_rust_, C_Cr_, and C_dl_—are larger in the Mg^2+^-containing seawater. The C_rust_ that represents the rust layer thickness shows the formation of a thin rust layer, due to the lower level of corrosion. The C_dl_ difference can be associated with ion absorption due to the Mg^2+^ ions in the seawater. The C_dl_ values between the blank and 0.7-wt.%-Cr steels, however, indicate a significant difference. This phenomenon means that the Cr-enrichment layer interrupts ionic absorption on the metal surface. Here, the C_Cr_, the thickness of the Cr-enrichment layer, represents the production of a thin Cr-enrichment layer in the Mg^2+^-containing seawater that is identified by the detected Cr element in Figure 1b and Figure 6b. The R_ct_ is a resistance value that prevents electron transfer from the metal layer to the rust layer, thereby indicating its relation to the metal corrosion rate. The larger R_ct_ values of the blank and 0.7-wt.%-Cr steels in the Mg^2+^-containing seawater indicate the suppression of the electron transfer compared with the Mg^2+^-free seawater.

### 2.4. Surface Analysis

**Figure 7:**
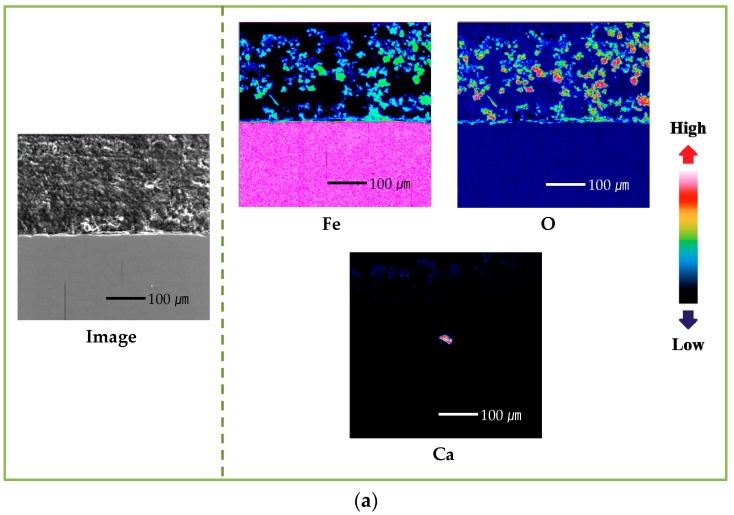

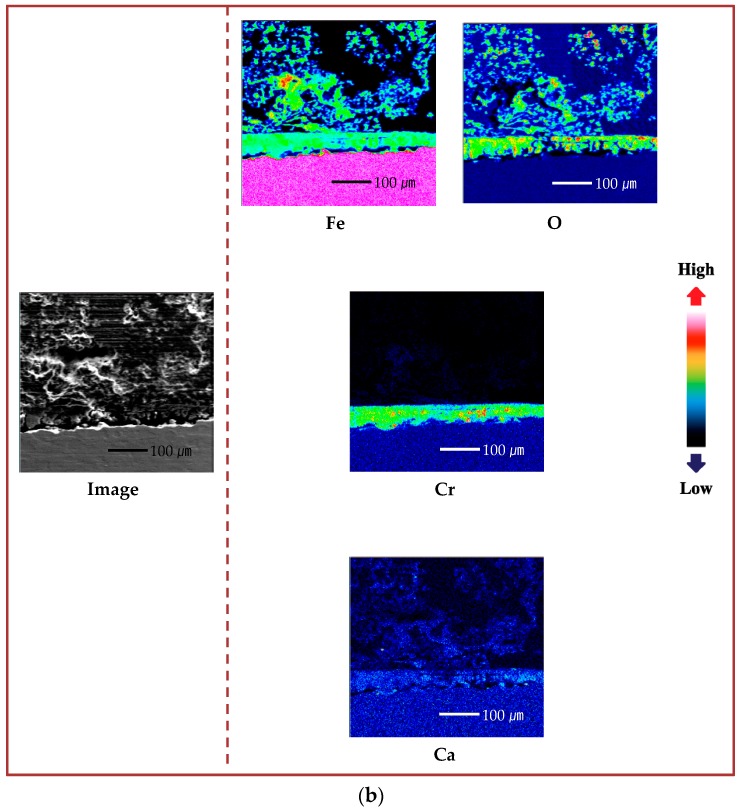
EPMA cross-sectional mapping results of specimens after 30 days immersion in seawater without Mg^2+^ ions: (**a**) blank steel and (**b**) 0.7-wt.%-Cr steel.

Figure 7 shows the cross-sectional EPMA-mapping results for the Mg^2+^-free seawater during the 30-day immersion. The tendencies, distributions, and concentrations of the Fe, oxygen (O), and Cr between the rust layer and the substrate are similar to those of the Mg^2+^-containing seawater, as shown in Figure 1. The 0.7-wt.%-Cr steel, with and without the Mg^2+^ ions, represents the Cr-enrichment layer, and the average thicknesses are approximately 25 μm and 45 μm, respectively. The Cr-enrichment layers are produced by the corrosion of the specimens, and the Cr^3+^ ions are redeposited onto the steel surface through reduction reactions [39]. Thus, the thicker thickness of the Cr-enrichment layer without the Mg^2+^ environment means that more corrosion occurred on the steel surface. This result means that the corrosion rate is more affected by the porosity than the thickness of the Cr-enrichment layer. The *R_p_* values that were measured at the 1- and 30-day immersions, which are shown in Table 1, indicate that the rust layer porosity affects the corrosion rate. In the Mg^2+^-containing seawater environment, both the blank and 0.7-wt.%-Cr steels increased the *R_p_* over time, whereas the *R_p_* decreased for both of the specimens in the Mg^2+^-free-seawater environment; this is because the base steel corrosion continues through the porous region of the rust layer.

**Figure 8:**
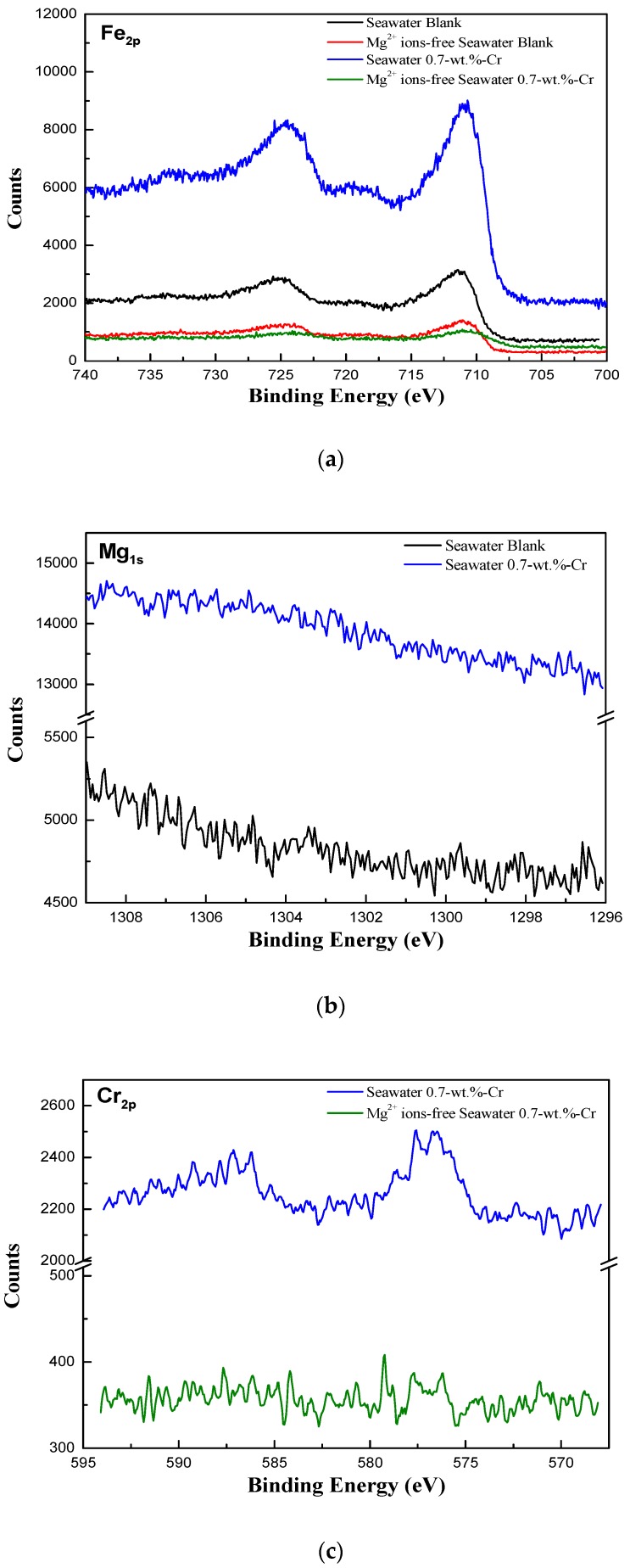
XPS spectra for the cross-sectional products of the specimens after 30 days immersion according to seawater conditions and alloying elements: (**a**) Fe, (**b**) Mg and (**c**) Cr.

To determine the chemical composition of the corrosion product after the 30-day immersion test, X-ray photoelectron spectroscopy (XPS) and X-ray diffraction (XRD) measurements were carried out. The local area near the Cr-enrichment layer was detected using XPS with the cross-section specimen, and the whole area was detected by XRD with a top-view specimen. Figure 8 shows the XPS spectra of the specimens. The Fe peaks were commonly found in all of the specimens, but the Mg and Cr peaks appeared in accordance with the experimental environment and the alloying element. The steel surface products that were obtained from the analysis of the XPS peaks are listed in Table 2. In Figure 8a, a proportional relationship is evident between the intensity values of the chemical compounds, such as magnetite (Fe_3_O_4_), iron(III) oxide (Fe_2_O_3_), and iron hydroxide (FeOOH), and the corrosion resistance. The increase in the amount of the Fe oxide products resulted in an enhanced corrosion resistance because the Fe oxide products act as the corrosion protection layer of the steels [34,40]. Similarly, in Figure 8b,c, the high intensity values of the Mg and Cr peaks mean that the effects of the Mg and Cr barriers react more significantly on the 0.7-wt.%-Cr steel in seawater. The difference in the intensity value is due to not only the amounts of the Mg(OH)_2_, chromium(III) oxide (Cr_2_O_3_), chromium hydroxide (CrOOH), and iron(II) chromite (FeCr_2_O_4_), but it is also owing to the formation of Mg and the Cr-containing product, magnesium(II) chromite (MgCr_2_O_4_). MgCr_2_O_4_, the structure of which is spinel, is known for its corrosion resistance improvement property [41,42,43,44]. It also acts as a p-type-semiconductor corrosive film that prevents the corrosion reaction [45]. Thus, the formation of MgCr_2_O_4_ increases the corrosion resistance of the 0.7-wt.%-Cr steel in seawater.

**Figure 9:**
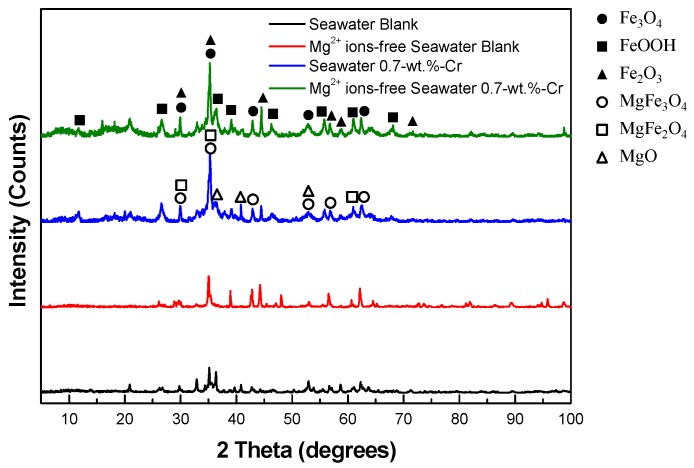
XRD spectra for the top-view products of specimens after 30 days immersion according to seawater conditions and alloying elements.

**Figure 10:**
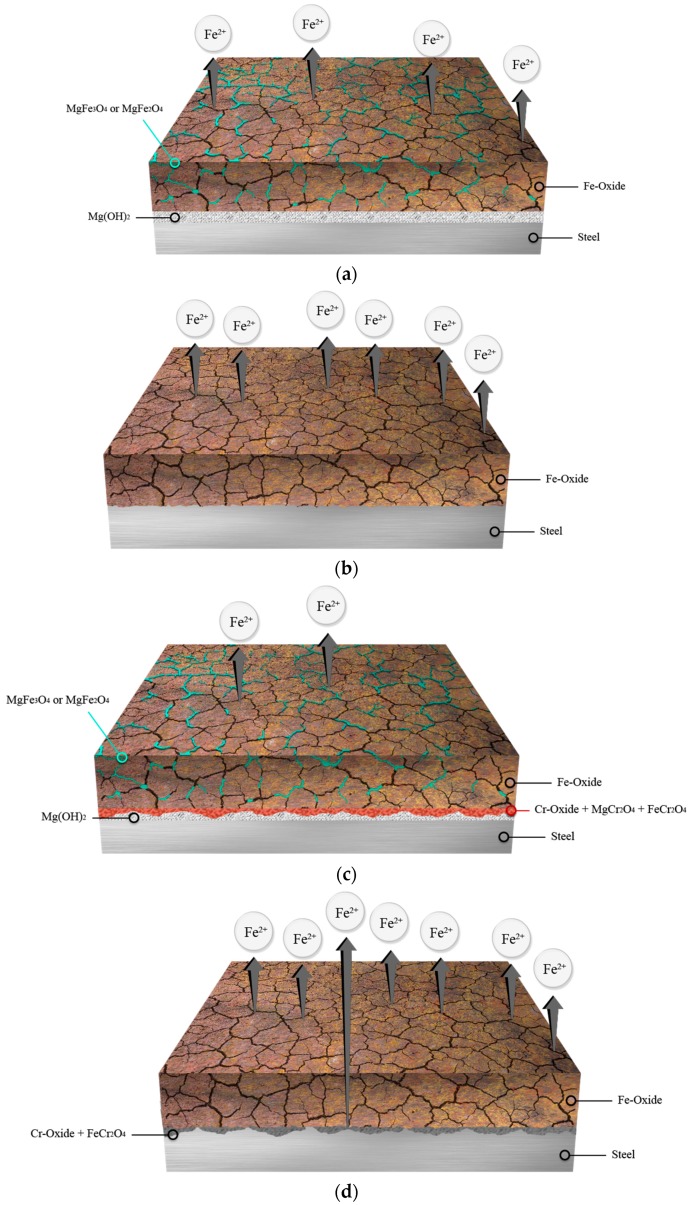
Schematic diagrams of corrosion products on specimens after 30 days immersion in seawater: (**a**) blank steel in seawater; (**b**) blank steel in Mg^2+^ ions-free seawater; (**c**) 0.7-wt.%-Cr steel in seawater and (**d**) 0.7-wt.%-Cr steel in Mg^2+^ ion-free seawater.

The XRD spectra of the specimens are shown in Figure 9. The Fe corrosion products show the same XPS results, but the other corrosion products, such as magnesium magnetite (MgFe_3_O_4_) and magnesium iron oxide (MgFe_2_O_4_), which are not shown in the XPS data, were detected. Wang et al. [46] reported that the existence of MgFe_2_O_4_ in the rust layer exerts a protective effect on weathering steel. Also, since the structure of MgFe_2_O_4_ is spinel, like that of MgCr_2_O_4_, this improves the corrosion resistance. The MgFe_2_O_4_ acts as an n-type-semiconductor corrosive film that works as a cation-selective rust layer [42,47,48]. Based on the surface analysis, the schematic diagrams of the corrosion products that affect the corrosion property are represented in Figure 10. Due to the corrosion barriers, such as MgFe_3_O_4_, the MgFe_2_O_4_ with Fe oxide, Cr oxide, FeCr_2_O_4_, and Mg(OH)_2_, the corrosion resistance of the 0.7-wt.%-Cr steel is the highest in seawater. In particular, for the 0.7-wt.%-Cr steel in seawater, the Cr oxide, FeCr_2_O_4_, and MgCr_2_O_4_ mixed layers contributed to the enhancement of the corrosion resistance. The corrosion resistance of the blank steel in seawater is the second highest, due to the MgFe_3_O_4_, the MgFe_2_O_4_ with Fe oxide, and Mg(OH)_2_. The corrosion resistance of the blank steel in the Mg^2+^-free seawater, which only comprises an Fe oxide corrosion barrier, is lower. Although the 0.7-wt.%-Cr steel in the Mg^2+^-free seawater consists of Fe oxide, Cr oxide, and FeCr_2_O_4_ barriers, the corrosion resistance is similar to that of the blank steel under the same seawater conditions. This phenomenon is due to the less-protective Cr oxide and FeCr_2_O_4_ layers and the galvanic-corrosion effect. On the steel surface, the Cr oxide and FeCr_2_O_4_ layers without the MgCr_2_O_4_ comprise the porosity property, as shown in the EIS results. Also, since the Cr oxide and the FeCr_2_O_4_ do not cover the steel surface perfectly, the Cr oxide behaves as the large cathode and the bare-steel surface behaves as the small anode. Thus, the large ratio of the cathode-to-anode surface area accelerated the galvanic-reaction corrosion of the 0.7-wt.%-Cr steel [28].

## 3. Materials and Methods

### 3.1. Materials and Test Condition

Table 3 summarizes the chemical compositions of the low-alloy steels that were used in the experiment. The specimens were produced by a thermomechanical control process (TMCP), which is a widely applied process for offshore structures and vessels, because of its superior mechanical properties, whereby both the rolling and cooling conditions are controlled [49,50,51]. Since the low-alloy steel is produced by vacuum melting, there is no contamination from the surroundings and the gas content of the steel is low. In addition, since the low-alloy steels were produced in small quantities to improve the product quality, the alloying elements were evenly distributed at the time of production. The plate of the low-alloy steels was cut into pieces of approximately 1 × 1 × 1.5 cm. The specimens were abraded with silicon carbide (SiC) papers, with grit sizes from 60 to 600, followed by rinsing with ethanol and distilled water, and finally drying was performed with a drying machine. The prepared specimen was immersed in the synthetic seawater at 60 °C and a pH of 8.2, under aerated conditions for 30 days; this temperature was selected because the inner part of the WBT can be reached by solar heat up to 60 °C [27,52]. The synthetic seawater solution was prepared based on the American Society for Testing and Materials (ASTM) standard D1141 [53]. Magnesium chloride (MgCl_2_) was excluded from the D1141 standard for the preparation of the Mg^2+^-free seawater, and the chlorine (Cl) concentration was adjusted with sodium chloride (NaCl).

### 3.2. Electrochemical Test

The EIS analysis and the LPR test were performed using the EG&G PAR VMP2 potentiostat/galvanostat (Princeton Applied Research, VMP2 & VMP2/Z multichannel potentiostats, BioLogic science instruments, Knoxville, TN, USA). To perform the electrochemical tests, the three-electrode electrochemical system consists of a low-alloy steel, two pure graphites, and a saturated calomel electrode (SCE) that served as the working, counter, and reference electrodes, respectively. The EIS analysis was carried out to calculate the corrosion rate and to observe the change in the rust layer with an amplitude of 10 mV, at frequencies ranging from 100 kHz to 1 mHz (or 10 mHz in the 1-day case). With the use of the Zsimpwin software (v3.2, Princeton Applied Research, Oak Ridge, TN, USA), the impedance plots were interpreted on the basis of the equivalent circuit, according to a suitable fitting procedure. The LPR tests were carried out at a potential sweep of 0.166 mV/s, from an initial potential of −20 mVocp, to a final potential of 20 mVocp. The polarization resistance was obtained from the slope of the potential versus the current-density curve.

### 3.3. Weight-Loss Test

Weight-loss measurements were performed according to the ASTM standard, G31 [54]. The lengths of three sides of rectangular specimens were measured up to two decimals. Every specimen was immersed in the synthetic seawater after a plastic wire hanging. After the immersion period of 30 days, the specimens were removed and then cleaned with a 10-min immersion in a 1000-mL solution that was composed of 3.5 g of hexamethylene tetramine (C_6_H_12_N_4_), 500 mL of hydrogen chloride (HCl), and balanced distilled water. These specimens were also degreased for 10 min in an ultrasonic cleaner with ethanol, followed by cleaning with distilled water and drying with N_2_, and then their final mass was measured to four decimal places. To improve the reliability, this experiment was performed repetitively in triplicate.

### 3.4. Surface Analysis

To investigate the effect of Cr and the relationship between Cr and the seawater cation, a surface analysis was carried out. The corroded surface features after the 30-day immersion in the 60 °C synthetic seawater, depending on the solution states, were observed using the JXA-8900R EPMA (JEOL, Tokyo, Japan). The chemical composition of the rust layer was identified using the ESCALAB 250 XPS instrument (Thermo Fisher Scientific, Waltham, MA, USA) with a monochromatic Al-K*α* energy source and the D/Max 2500 V PC XRD instrument (Rigaku Corporation, Tokyo, Japan) with a scan rate of 3°/min from 5–100°.

## 4. Conclusions

This study investigated the synergic effect of the alloying element, Cr, and the environment element, Mg^2+^, on the corrosion of low-alloy steels in synthetic seawater at 60 °C, using EIS, LPR tests, weight-loss tests, and a surface analysis. Based on the previously presented results, the following conclusions can be drawn:

1. From the EPMA results in the seawater, the 0.7-wt.%-Cr steel represents the Cr-enrichment layer, and the Mg^2+^ ions in the seawater are also concentrated on the Cr-enrichment layer.
2. The EIS analysis, LPR tests, and weight-loss tests, with and without the Cr-alloying element and the Mg^2+^-containing seawater, revealed the following order for the average corrosion rates of the steels: 0.7-wt.%-Cr steel without the Mg^2+^ ions ≈ blank steel without the Mg^2+^ ions > blank steel with the Mg^2+^ ions > 0.7-wt.%-Cr steel with the Mg^2+^ ions. The Cr-alloying element only improved corrosion resistance in the Mg^2+^-containing seawater, and the reason is not only the Mg^2+^-containing effect, but also the barrier effect between the Mg^2+^, Fe^2+^, and Cr^3+^ ions.
3. The EIS interpretation of the blank and 0.7-wt.%-Cr steels suggests that both of the steels under the seawater condition show a rust layer, with a relatively nonporous corrosion on the steel surface.
4. The XPS and XRD analyses showed that the rust layer of the 0.7-wt.%-Cr steel in the seawater comprises effective protection barriers, such as MgFe_3_O_4_, MgFe_2_O_4_, MgCr_2_O_4_ with Fe oxide, Cr oxide, FeCr_2_O_4_, and Mg(OH)_2_. Also, the high-porosity condition of the Cr oxide layer was changed to a low-porosity condition.

## Figures and Tables

**Table 1:** EIS parameters of the specimens immersed in 60 °C seawater for 1 day and 30 days under an aerated condition.

Specimen	Period	R_s_ (Ω∙cm^2^)	Constant Phase Element 1		R_rust_ (Ω∙cm^2^)	Constant Phase Element 2		R_Cr_ (Ω∙cm^2^)	Constant Phase Element 3		R_ct_ (Ω∙cm^2^)	*R_p_* (Ω∙cm^2^)
			C_rust_ (F/cm^2^)	*n* (0–1)		C_Cr_ (F/cm^2^)	*n* (0–1)		C_dl_ (F/cm^2^)	*n* (0–1)		
Blank steel (Seawater)	1 day	2.286	-	-	-	-	-	-	0.58 × 10^−4^	0.7818	400.9	400.9
	30 days	5.349	4.23 × 10^−3^	0.7643	30.51	-	-	-	28.20 × 10^−3^	0.4801	632.7	663.21
				(*α* = 21.213)								
Blank steel (Mg^2+^ ions-free Seawater)	1 day	1.93	-	-	-	-	-	-	0.38 × 10^−4^	0.7991	406.6	406.6
	30 days	4.164	3.48 × 10^−3^	0.7463	23.26	-	-	-	13.76 × 10^−3^	0.5292	310.7	333.96
				(*α* = 22.833)								
0.7-wt.%-Cr steel (Seawater)	1 day	2.202	-	-	-	-	-	-	0.65 × 10^−4^	0.7806	420.7	420.7
	30 days	3.456	3.68 × 10^−3^	0.3949	3.64	0.68 × 10^−3^	0.8063	32.88	6.20 × 10^−3^	0.6321	769.3	805.82
							(*α* = 17.433)					
0.7-wt.%-Cr steel (Mg^2+^ ions-free Seawater)	1 day	2.077	-	-	-	-	-	-	0.75 × 10^−4^	0.7480	415.7	415.7
	30 days	5.563	1.59 × 10^−3^	0.5022	9.51	0.45 × 10^−3^	0.7646	55.95	1.19 × 10^−3^	0.7432	262.3	327.76
							(*α* = 21.186)					

**Table 2:** Analysis of the XPS peaks for the surface of the specimens.

Analyses of the XPS Spectra	Product	Binding Energy (eV)
Spectrum of Fe_2p_	FeOOH	711.5, 724.3
	Fe_2_O_3_	711.0, 724.0, 710.8
	Fe_3_O_4_ (Fe^2+^)	708.3
	Fe_3_O_4_ (Fe^3+^)	710.2
Spectrum of Mg_1s_	Mg(OH)_2_	1302.7
	MgO	1303.9
Spectrum of Cr_2p_	Cr_2_O_3_	576.5, 576.8, 587.4
	CrOOH	576.8, 577
	FeCr_2_O_4_	576.0
	MgCr_2_O_4_	576.4

**Table 3:** Chemical compositions of low-alloy steels (wt.%).

Specimen	Fe	C	Si	Mn	P	S	Nb	Ti	Cr
Blank steel	Balance	0.07	0.3	1	0.012	0.003	0.01	0.015	-
0.7-wt.%-Cr steel	Balance	0.07	0.3	1	0.012	0.003	0.01	0.015	0.7
